# Tuberculosis incidence in a cohort of individuals infected with human T-lymphotropic virus type 1 (HTLV-1) in Salvador, Brazil

**DOI:** 10.1186/s12879-016-1428-z

**Published:** 2016-09-19

**Authors:** Maria Fernanda Rios Grassi, Normeide Pedreira dos Santos, Monique Lírio, Afrânio Lineu Kritski, Maria da Conceição Chagas Almeida, Leonardo Pereira Santana, Noilson Lázaro, Juarez Dias, Eduardo Martins Netto, Bernardo Galvão-Castro

**Affiliations:** 1 Laboratório Avançado de Saúde Pública, Fundação Oswaldo Cruz-Bahia (FIOCRUZ), Rua Waldemar Falcão, 121, Candeal, CEP: 40296-710 Salvador, Bahia Brazil; 2 Escola Bahiana de Medicina e Saúde Pública, Salvador, Bahia Brazil; 3 Hospital Universitário professor Edgar Santos, Universidade Federal da Bahia, Salvador, Bahia Brazil; 4 Programa Acadêmico de Tuberculose, Faculdade de Medicina, Universidade Federal do Rio de Janeiro, Rio de Janeiro, Rio de Janeiro Brazil; 5 Laboratório de Epidemiologia, Fundação Oswaldo Cruz- Bahia, Salvador, Bahia Brazil; 6 Departamento de Vigilância Epidemiológica, Secretaria da Saúde do Estado da Bahia, Salvador, Bahia Brazil

**Keywords:** Human T-lymphotropic virus type 1, HTLV-1, Tuberculosis, Incidence, Relative risk

## Abstract

**Background:**

Few reports have investigated the association between human T-lymphotropic virus type 1 (HTLV-1) and tuberculosis (TB) in countries where both infections are endemic. This study estimates the incidence of TB in a cohort infected with HTLV-1, compared with non-infected individuals, over a ten-year period.

**Methods:**

Retrospective cohort study involving the cross-matching of records of individuals for whom a HTLV serology was performed at a referral center for HTLV (CHTLV) with a database of TB cases from Sinan—the Information System on Diseases of Compulsory Declaration between 2002 and 2012.

**Results:**

From a cohort of 6,495 individuals, 1,711 were infected with HTLV-1. A total of 73 TB cases occurred during the study period: 33 HTLV-1-infected patients and 40 uninfected individuals. The incidence density for TB in the HTLV-1 infected group was 3.3 person-years per 1,000 individuals and 1.1 person-years per 1,000 individuals in the group HTLV-1 uninfected group. The relative risk of developing TB in the group of patients infected with HTLV-1 was 2.6 (CI 95 % 1.6–4.2) in comparison with HTLV-1 uninfected group. Compared to individuals with isolated TB, those in the HTLV-1 infected group who had TB were older (*p* = 0.005) and had lower education levels (*p* = 0.02). No differences were observed with respect to the clinical/radiological presentation, nor in the outcome of TB and prevalence of HIV infection, when comparing among the HTLV-1-infected and uninfected groups.

**Conclusions:**

Patients infected with HTLV-1 are more susceptible to TB. The epidemiological characteristics of HTLV-1/TB subjects and those infected with TB overlap.

## Background

Human T-lymphotropic virus type 1 (HTLV-1) infects around 10 million people worldwide, most of whom are concentrated within endemic areas in developing countries. Brazil is one of the largest endemic areas of HTLV-1 in the world [1]. This virus is also recognized as the etiological agent of adult T-cell leukemia/lymphoma (ATLL) [2], HTLV-1-associated myelopathy/tropical spastic paraparesis (HAM/TSP) [3] and HTLV-1-associated uveitis [4], which, together, affect between 5–10 % of all HTLV-1 infected individuals [5]. Other clinical manifestations, such as arthritis, overactive bladder, myositis and bronchiectasis are also frequently present in infected individuals, attesting to the systemic nature of this viral infection [6–8].

Several reports have indicated that HTLV-1 may promote some degree of immunosuppression, as an association has been observed between HTLV-1 and Norwegian scabies [9, 10], disseminated strongyloidiasis [11] and infective dermatitis [12]. Moreover, healthy HTLV-1 carriers exhibit a suppressed response to the tuberculin skin test (TST) [13] and their cells fail to respond to recall antigens in vitro, such as recombinant purified protein derivative (PPD) [14]. Previous studies that have investigated the association between tuberculosis (TB) and HTLV-1, which were conducted in areas endemic for both infections, found a higher prevalence of HTLV-1 infection in patients diagnosed with tuberculosis (TB) in comparison to these patients’ relatives or control groups [15–19]. Conversely, other authors did not detect a significant prevalence of HTLV-1 in TB groups compared with controls [20, 21]. In addition, scattered reports have shown contradictory results with respect to the clinical outcomes of patients infected with both HTLV-1 and TB. While some of these reports described higher mortality in co-infected patients compared to those with isolated TB [22, 23], another found no differences regarding mortality or clinical characteristics [24].

Salvador, the capital of the state of Bahia, located in Northeastern Brazil, has been described as the epicenter of the HTLV-1 endemic in the country, with a high prevalence of infection in blood donors (1.35 %) [25], pregnant women (0.88 %) [26] and in the general population (1.74 %) [27]. The state of Bahia ranks third in terms of number of TB cases nationwide, and the incidence in Salvador reaches 62.3/100,000 inhabitants [28]. Taking into account the fact that both infections typically affect economically vulnerable populations, we hypothesized that the incidence of TB may be higher in individuals infected with HTLV-1 in areas in which both infections are endemic. Accordingly, the present study aimed to estimate the incidence of TB in a cohort infected with HTLV-1, compared with non-infected individuals, over a ten-year period.

## Methods

### Area and study population

This retrospective cohort study involved the cross-matching of records of individuals for whom HTLV serology was performed at a referral center for HTLV (CHTLV) of the Bahiana School of Medicine and Public Health (EBMSP), in Salvador – Brazil, between 2002 and 2012. CHTLV is a free public outpatient clinic that has provided comprehensive care to HTLV-infected individuals and their relatives since 2002. Serological testing for HTLV-1/2 was conducted for 6,620 individuals at CHTLV throughout the period of study. This population consisted mainly of blood donors, pregnant women and patients with neurological symptoms, as well as their families, who were referred by blood banks, prenatal physicians or clinicians from the public health system. All individuals who underwent serologic testing for HTLV during the period of the study were included. Individuals with indeterminate serology, as well as those who tested positive for HTLV-2 and whose date of testing was not recorded, were excluded. To perform HTLV-1 diagnosis, all individuals were screened using ELISA (Cambridge Biotech Corp., Worcester, MA) and positive tests were subsequently submitted to Western Blot analysis (HTLV blot 2.4, MP Diagnosis, Singapore) to confirm infection. HTLV-1/2 co-infection was determined using WB, in accordance with manufacturer’s instructions: i) HTLV-1: reactivity to GAG (p19 with or without p24) and two ENV (GD21 and rgp46-I) proteins; ii) HTLV-2: reactivity to GAG (p24 with or without p19) and two ENV (GD21 and rgp46-II) proteins. PCR was used only in cases in which HTLV serology was indeterminate; however, these patients with this serological status were excluded. The EBMSP Institutional Review Board approved the present study.

### Study design and data collection

This retrospective cohort study was based on patient records from CHTLV and on the TB registry from the Information System on Diseases of Compulsory Declaration (Sistema de Informação de Agravos de Notificação—Sinan). TB notification has been mandatory in Brazil since 1998. The number of TB cases reported by the SINAN database was 79,942 in Bahia state between January 2002 and May 2012. After the exclusion of duplicates (5,211) or corrupted names (3), the final number of TB cases was 74,454 (93.1 %). Case of tuberculosis was defined as a patient who experienced respiratory symptoms and had a smear-positive test for *Mycobacterium tuberculosis*. TB diagnosis was also considered in presence of a positive culture or clinical history of TB associated with complementary tests, for example a suggestive chest X-ray with heterogeneous opacity of the lung parenchyma, cavitation, nodules or consolidations [29]. Search for TB case notifications was done in Sinan database using SQL (Structured Query Language for use in relational databases) to identify patients with records at both CHTLV and in the Sinan registry by employing a “linkage” strategy (record linkage). The key variables considered were patient full name, date of birth and mother’s full name. Following patient identification, the epidemiological and clinical variables of interest were: age at time of TB diagnosis, gender, education level, admission status (new case, relapse), result of chest X-ray, TST result, clinical form of tuberculosis (pulmonary or extrapulmonary), HIV status and TB treatment outcome. All data analyzed were anonymized.

### Statistical analysis

For purposes of analysis, subjects were classified into two groups according to the results of serology for HTLV-1. The independent variable was HTLV-1 and the outcome was the diagnosis of TB. The data were expressed as mean and standard deviation, proportions, measures of central tendency and interquartile range. In order to test the differences between means Student t was used and for proportions, the chi-square test. As measures of frequency, incidence density for both groups was calculated: coinfected (HTLV-1/TB) and isolated TB and the relative risk of TB in those infected with HTLV-1. The date of the serology was considered as the moment of inclusion in the cohort. The TB disease incidence density in both HTLV-1-infected and uninfected groups was calculated as the number of new-TB cases per 1000 person-years of follow-up. The analysis was further stratified by the age and the crude incidence density was adjusted using Mantel-Hansel test. Then, a multivariate analysis using the Poisson model was performed considering person-time and age and sex as potential confounding. A *P* < 0.05 was considered statistically significant. All analyzes were performed using STATA 13 statistical software (Stata Corp, College Station, TX, USA).

## Results

**Fig. 1:**
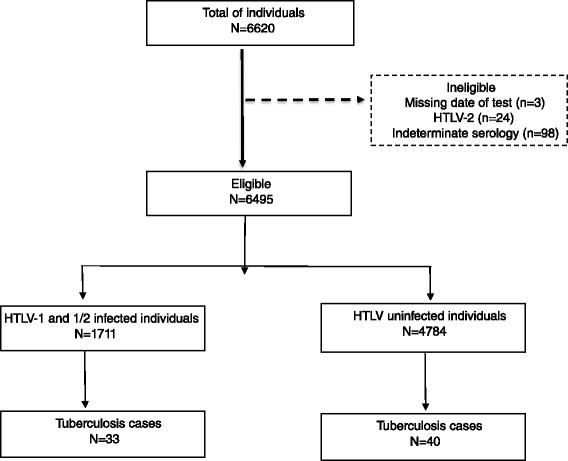
Flow diagram of studied patients

From January 2002 to April 2012, 6,620 individuals were tested for HTLV-1/2 at the CHTLV, 125 of whom were excluded (Fig. 1). The number of individuals infected with HTLV-1 was 1,711 (1,703 had HTLV-1 and eight were co-infected with both HTLV-1 and HTLV-2), while 4,784 had negative HTLV serology. The mean age of individuals either infected or uninfected with HTLV-1 was 43.5 ± 16.6 years and 33.3 ± 18.0 years (*p* = 0.000), respectively. The percentage of females was 82.9 % in the HTLV-1 infected group and 67.5 % in the uninfected group (*p* = 0.000). The final sample consisted of 6,495 individuals, with a total of 46,634 person-years of follow-up (mean 6.4 ± 2.7 years). A total of 73 cases of TB occurred in members of the cohort during the period studied: 33 among patients infected with HTLV-1 and 40 in the uninfected group. No TB cases were found among patients coinfected with both HTLV-1 and HTLV-2. About half the individuals (52 %) received a positive TB diagnosis prior to being diagnosed with HTLV-1, with a mean time difference between TB and HTLV-1 diagnoses of -1 ± 4 years. The overall incidence density for the HTLV-1/TB group was 3.3 person-years per 1,000 individuals and 1.1 person-years per 1,000 individuals in the non-infected control group. The overall relative risk of TB in the HTLV-1-infected group was 2.6 (CI 95 % 1.6–4.2). When the sample was stratified by age, a significantly higher relative risk of TB (3.4, CI 95 % 1.6–7.2) was found only in individuals aged 31 to 50. The Poisson model returned a relative risk of TB, adjusted for age and sex, of 2.5 (IC95% 1.6–4.1) for HTLV-1 infected individuals, in comparison with uninfected persons (Table 1).

**Table 1:** Tuberculosis incidence density and relative risk stratified by age

	HTLV-1-infected group			HTLV-1 uninfected controls				
	TB cases	Person year	ID/1000	TB cases	Person year	ID/1000	RR	CI 95 %
0 to 30ys	4	2,254	1.8	19	19,845	1.1	1.9	0.6–5.5
31 to 50ys	15	4,477	3.4	12	12,058	1.0	3.4	1.6–7.2
>50ys	14	3,263	4.3	9	4,737	1.9	2.3	1.0–5.2
All	33	9,994	3.3	40	36,640	1.1	2.6	1.6–4.2

Age at the TB diagnosis. HTLV-1 = Human T-lymphotropic virus type 1; TB = tuberculosis; ID = Incidence density RR- relative risk adjusted using Mantel-Hansel test CI: confidence interval

The majority of individuals in the HTLV-1/TB group (54.8 %) had not completed primary school, while this proportion was 21.7 % in the isolated TB group (*p* = 0.009), (Table 2).

**Table 2:** Epidemiological characteristics of 73 patients with tuberculosis diagnosis according to HTLV-1 infection

Characteristics	HTLV-1-infected		HTLV-1-uninfected		
	*n* = 33	%	*n* = 40	%	*p* value*
Age (years) Mean ± SD	49.7 ± 16.4		38.6 ± 15.9		0.005
Female	22	66.7	23	57.5	0.43
Educational level					0.009
Incomplete elementar	17	54.8	2	21.7	
Completed elementar	10	32.3	7	30.4	
High school graduate	4	12.9	11	47.8	

Age at the TB diagnosis. HTLV-1 = Human T-lymphotropic virus type 1; TB = tuberculosis; *Pearson Chi square, *P* < 0.05

Table 3 delineates the clinical characteristics of all individuals with TB. The frequency of chest X-ray suggestive of TB was similar in HTLV-1 infected individuals and those with isolated TB. The proportion of patients that had a recurrence of TB, and/or those who abandoned treatment and then subsequently resumed it, was slightly higher in individuals infected with HTLV-1 (21.2 %) compared to the HTLV-uninfected group (12.5 %), yet no statistical significance was detected (*p* = 0.09). The clinical presentations of TB (pulmonary/extra pulmonary) were similar in all individuals. No significant differences in TB outcomes (cure, treatment dropout or mortality) were observed when comparing the HTLV-1 infected and uninfected groups, although two HTLV-1-infected individuals died due to TB. TST was performed in 21.2 % of the individuals infected with HTLV-1 and in 30 % of individuals with TB. The proportion of reactive results ≥10 mm was 75.1 % (4 of 7) in the group infected with HTLV-1 and 83 % (10 of 12) in the TB-only group. HIV serology was positive for 6.0 % of the individuals infected with HTLV-1, versus 12.5 % with isolated TB (*p* = 0.45).

**Table 3:** Characteristics and outcomes of tuberculosis in patients infected with HTLV-1 compared to uninfected

Variables associated to TB	HTLV-infected (*n* = 33)		HTLV-1-uninfected (*n* = 40)		p value*
	N	%	n	%	
Admission status					0.09
New case	19	57.6	32	80	
Relapse/readmission	7	21.2	5	12.5	
Chest X ray					0.55
Suspect^a^	28	84.9	30	75	
Normal	2	6.0	5	12.5	
Others	3	9.1	5	12.5	
Clinical form of TB					0.71
Pulmonary	28	84.8	31	77.5	
Extra-pulmonary	5	15.2	7	17.5	
Both	-	-	2	5	
TB outcome					0.54
Healing/diagnosis change	22	66.7	29	72.5	
Abandonment/death/transfer	2	6.1	4	10	
Other	9	27.3	7	17.5	
TST					ND
Non reactor	2	6.1	1	2.5	
>5 and <10 mm	1	3	1	2.5	
>10 mm	4	12.1	10	25	
Not performed	26	78.8	28	70	
HIV serology					0.45
Positive	2	6.0	5	12.5	
Negative	29	89	34	85	
Not performed	2	6.0	1	2.5	

HTLV-1 = Human T-lymphotropic virus type 1; TB = Tuberculosis; TST = tuberculin skin test; HIV = human immunodeficiency virus; ^a^heterogeneous opacity of the lung parenchyma, cavitation, nodules, consolidations. *Pearson Chi square, *P* < 0.05

## Discussion

This is the first retrospective cohort study to assess the risk of developing TB in HTLV-1-infected individuals residing in a high TB-burden country. The results herein indicate that the risk of developing TB in HTLV-1-infected individuals is almost three times greater than in uninfected controls over a 10-year period of follow-up. This finding is particularly relevant because all studies addressing the association between HTLV-1 and TB to date have been carried out in populations that were previously diagnosed with TB. Moreover, several of these studies reported contradictory results; i.e. some found an increased prevalence of TB in HTLV-1 infected individuals [15–19], while others failed to confirm this association [20, 21]. The present study found an overall TB incidence density of 3.3 per 1,000 person-years in the HTLV-1-infected group. A higher risk of TB was found mainly in individuals aged 31 to 50 years. These findings corroborate a case control study carried out in Salvador-Bahia that found a three times greater risk of HTLV-1 infection in patients diagnosed with TB, compared to those without TB [18]. In HTLV-1 uninfected group, the incidence density reached 1.1 per 1,000 person-years, which represents a higher incidence than that found in the general population. The uninfected group consisted of blood donors, which are generally healthy, but also pregnant women, patients with neurological symptoms and relatives of HTLV-1-infected individuals for whom a serological HTLV-1 test had been recommended. Thus, this group is not representative of general population. Moreover, individuals were mostly from Salvador, which presented an annual TB incidence of 62.3/100,000 inhabitants in general population in 2012 [30], compared with a national incidence of 35.4/100,000 [31]. Taken together, these data strongly suggest that an association exists between HTLV-1 and TB in areas in which both infections are endemic.

Differences in sociodemographic characteristics were observed between patients with HTLV-1+/TB versus the group with TB alone. The epidemiological profile of patients coinfected with HTLV-1/*M. tuberculosis* was consistent with the epidemiological characteristics described by a population-based study conducted in Salvador to determine HTLV-1 infection prevalence [27]: a mean age of 50 years at time of TB diagnosis, lower educational level, predominance of females. Dourado et al. reported a 1.76 % overall prevalence of HTLV-1, with a higher prevalence observed in women (9.3 %) over 51 years compared to men (6.3 %), as well as in individuals with less education and lower income. Although lower income is also associated with increased susceptibility to TB in Brazil [32], this disease is more frequently diagnosed in men under 50. In addition, TB burden varies according to geographic region. Bahia is the third state in terms of absolute number of cases, and Salvador has the second highest incidence of TB among the capitals in northeastern Brazil [28]. These data indicate that HTLV-1-infected individuals who are impoverished and possess low educational levels may suffer from greater susceptibility to TB.

Clinical features of TB infection between the two groups revealed that the clinical presentations (pulmonary or extra-pulmonary) and the number of chest-X-rays suggestive of TB were similar. Although a greater number of individuals in the coinfected group had higher rates of TB recurrence or returned to treatment after abandonment, this difference was not statistically significant. A positive TST result was more frequently observed among individuals serologically negative for HTLV-1 (25 %), compared with 12.1 % of patients with HTLV-1/TB, which may indicate some degree of immunosuppression in response to antigens of *M. tuberculosis*. However, this result should be carefully interpreted since only one-third of the subjects in both groups had TST results registered in their medical records.

Nonetheless, a decreased response to TST was reported in a cohort of patients infected with HTLV-1 in Japan [16], and a decreased proliferative response to purified protein derivate (PPD) was also described in asymptomatic individuals infected with HTLV-1 in Salvador-Brazil [17]. Conversely, another study did not find any differences in the number of patients with a positive TST result when comparing individuals with HTLV-1/TB or isolated TB [24]. Regarding HIV-1 serological status, the percentage of individuals co-infected by this virus in the HTLV-1/TB group was half that of the group with TB alone (12 %), and similar to the overall rate of HIV found by testing patients with tuberculosis [30]. Therefore, increased susceptibility to TB in HTLV-1-infected individuals was found to have no association with HIV-infection. Relevant data regarding sputum smears (taken at the time of diagnosis and after the initiation of treatment) and *M. tuberculosis* cultures were lacking in the majority of patient records.

The underlying cause surrounding the increased susceptibility to TB observed in individuals infected with HTLV-1 remains unknown. Nonetheless, it is possible that immunological changes induced by HTLV-1 might also affect TB susceptibility. The protective immune response against *M. tuberculosis* has been shown to be dependent on INF-γ-activated macrophages and, interestingly, HTLV-1 induces the proliferation and activation of CD4^+^ T-cells, especially type 1, which consequently produce elevated levels of INF-γ and TNF [33, 34]. It has also been suggested that HTLV-1 infected patients may, paradoxically, have an increased susceptibility to TB due to impaired TNF-α production in response to *M. tuberculosis* antigens [24]. Further studies evaluating the immune response in a larger sample of individuals coinfected with HTLV-1/M. tuberculosis should be conducted.

The present study is limited by the fact that information regarding the status of diabetes, alcoholism and smoking habits, which are all recognized risk factors for TB, was not available for all individuals. In addition, it was not possible to determine how many patients with TB and HIV infection (in both HTLV-1 infected or uninfected groups) were under antiretroviral treatment. As such, we cannot exclude the possibility that HTLV-1-infected patients were more prone to develop TB as a result of these comorbidities. It is also possible that a portion of the cohort, which was regularly followed due to HTLV-1 infection, presented increased rates of TB due to regular surveillance. As TB and HTLV-1 were diagnosed in different medical settings, 51 % (17/33) of HTLV-1-infected individuals received their TB diagnosis in a primary care setting, while 67 % (27/40) of HTLV-1-infected individuals received a TB diagnosis in secondary/tertiary medical units. In addition, in over half of these coinfected patients, HTLV-1 serological status was not known at the time of TB diagnosis.

Unfortunately, it was not possible to determine the successful rate of linkage. However, since these databases were not very large, the results from both databases were also visually verified. Thus, we believe that the linkage rate was likely high. We were also unable to determine the number of persons lost to follow-up during the study period. It is possible that unreported cases resulted from a myriad of factors, including loss of contact, death, change-of-address, etc. Moreover, it is interesting to note that the World Health Organization [35] estimates that 82 % of the true TB cases are captured by the SINAN database in Brazil. Even under the assumption of a 100 % accurate report rate among HTLV-1-infected individuals and 82 % among uninfected controls, the incidence would still be 3.3 % among HTLV versus 1.3 % (instead 1.1 %) among HTLV negative individuals, which, nonetheless, remains significantly different.

## Conclusions

In summary, the risk of developing TB was found to be almost three times greater in people living with HTLV-1 than among those without this viral infection. HTLV-1/TB individuals were more commonly female, aged 50 years or older, and had lower income and educational levels than individuals infected only with TB. These epidemiological features overlap those present in the profile of HTLV-1 infection. Each infection is strongly associated with greater social vulnerability, reinforcing the neglected characteristic of both diseases. The results presented herein indicate that, in countries endemic for both infections, health professionals should actively monitor HTLV-1 infected patients for respiratory symptoms.

## Abbreviations

CHTLV: HTLV Reference Center (Unit Care for patients infected with the virus)
HTLV-1: Human T-lymphotropic virus type 1
PPD: Purified protein derivative
SINAN: Information System on Diseases of Compulsory Declaration
TB: Tuberculosis
TST: Tuberculin skin test
